# The Influence of Bi_2_O_3_ Nanoparticle Content on the γ-ray Interaction Parameters of Silicon Rubber

**DOI:** 10.3390/polym14051048

**Published:** 2022-03-06

**Authors:** Mahmoud I. Abbas, Ahmed M. El-Khatib, Mirvat Fawzi Dib, Hoda Ezzelddin Mustafa, M. I. Sayyed, Mohamed Elsafi

**Affiliations:** 1 Physics Department, Faculty of Science, Alexandria University, Alexandria 21511, Egypt; mabbas@physicist.net (M.I.A.); elkhatib60@yahoo.com (A.M.E.-K.); mirvatdib2018@gmail.com (M.F.D.); 2 Khalifa Medical Center, Abu Dhabi W13-01, United Arab Emirates; hezzeddin68m@gmail.com; 3 Department of Physics, Faculty of Science, Isra University, Amman 11622, Jordan; dr.mabualssayed@gmail.com; 4 Department of Nuclear Medicine Research, Institute for Research and Medical Consultations, Imam Abdulrahman bin Faisal University, Dammam 31441, Saudi Arabia

**Keywords:** silicon rubber, nano-Bi_2_O_3_, *LAC*, RPE, HVL

## Abstract

In this study, synthetic silicone rubber (SR) and Bi_2_O_3_ micro- and nanoparticles were purchased. The percentages for both sizes of Bi_2_O_3_ were 10, 20 and 30 wt% as fillers. The morphological, mechanical and shielding properties were determined for all the prepared samples. The Linear Attenuation Coefficient (*LAC*) values of the silicon rubber (SR) without Bi_2_O_3_ and with 5, 10, 30 and 30% Bi_2_O_3_ (in micro and nano sizes) were experimentally measured using different radioactive point sources in the energy range varying from 0.06 to 1.333 MeV. Additionally, we theoretically calculated the *LAC* for SR with micro-Bi_2_O_3_ using XCOM software. A good agreement was noticed between the two methods. The NaI (Tl) scintillation detector and four radioactive point sources (Am-241, Ba-133, Cs-137 and Co-60) were used in the measurements. Other shielding parameters were calculated for the prepared samples, such as the Half Value Layer (HVL), Mean Free Path (MFP) and Radiation Protection Efficiency (RPE), all of which proved that adding nano-Bi_2_O_3_ ratios of SR produces higher shielding efficiency than its micro counterpart.

## 1. Introduction

In medical facilities, such as hospitals, clinics, outpatient care centers, radiological centers and dental facilities, where ionizing radiation is widely utilized, planning is compulsory to protect patients and medical staff who are usually exposed to different types of radiation. For this reason, it is important to use radiation protection materials, whether or not these materials are worn, such as eyeglasses, neck guards or an apron [1,2,3,4]. Moreover, it is important to utilize specific materials to insulate the walls of the medical facilities in order to prevent radiation leakage into the surrounding environment. This applies not only to the medical facilities, but also all facilities that utilize gamma radiation or X-ray, such as universities and research laboratories, nuclear power plants, and factories [5,6].

The attenuation properties for the radiation protection medium must be accurately known when planning to design any facility that uses gamma rays and X-rays, so that appropriate protection is provided for patients, workers, visitors, and the surrounding environment [7,8,9]. The radiation protection properties of a medium depend on its density as well as the chemical composition of the medium’s constituent materials. Thickness is also considered as another factor that affects the shielding properties of a given medium. The traditional materials that are practically used in radiation protection applications have several drawbacks.

Some of these materials are expensive and some are heavy, and this limits their use in practical applications. For example, tungsten has a higher number of attenuation factors than lead; it also has a high cost and this prevents tungsten from being widely used in real applications. Recently, researchers turned to the production of radiation protective clothing that is characterized by its low cost, light weight, easy use, comfort and, most importantly, its protection of workers in the field of ionizing radiation [10,11,12]. In this regard, some additives are usually added to the flexible materials to fabricate protective garments, such as aprons or curtains. In practical applications, vinyl, polymers and rubbers are one of the most widespread materials used as matrix materials to obtain flexible protection materials, while bismuth, tungsten and antimony powders are used as additives. The importance of such additives is to increase the possibilities of the photon interactions with the atoms of the prepared flexible protective materials.

As is known, the polymers and plastics have a relatively low density and this, to a certain degree, produces their ability to attenuate photons. Therefore, these plastic materials are usually used to attenuate low-energy radiation [13,14,15]. In order to increase its density and thus improve its shielding performance, especially if it is exposed to photons of medium energy, bismuth is used [16,17]. Silicone rubber (SR) is an important matrix material that has good elasticity features. In the past few years, some researchers used silicone rubber to develop flexible radiation shielding materials. Kameesy et al. [18] fabricated SR sheets filled with four concentrations of PbO. They experimentally evaluated the radiation attenuation factors for the prepared SR-PbO campsites using different radioactive sources. They found that adding PbO to the SR enhances the physico-mechanical features. Gong et al. [19] fabricated a novel radiation protection composite based on methyl vinyl silicone rubber. The authors found that when benzophenone is added to the matrix, a notable enhancement in the radiation resistance occurs. Based on their results, the transmission of the photon with energy of 0.662 MeV through a sample thickness of 2 cm is only 0.7. Özdemir and Yılmaz [20] prepared a mixed radiation shielding via 3-layered polydimethylsiloxane rubber composite. The three layers were composed of hexagonal boron nitride, B_2_O_3_ and Bi_2_O_3_. They developed a shielding material that possesses a lead equivalent thickness of 0.35 mm Pb. Chai et al. [21] prepared new flexible shielding material using methyl vinyl silicone rubber. They used zinc borate, B_4_C and hollow beads as filler materials. They evaluated the neutron shielding performance of their flexible material of the thermal neutron transmission technique with the help of an Am–Be radiation source. However, even though different research groups studied the SR as flexible shielding materials, there is still an urgent need for the further development of novel flexible materials using Bi_2_O_3_ as a filler in nano and micro sizes. Therefore, in this study, we develop a new flexible material against X-ray and γ-ray photons based on Bi_2_O_3_ nanoparticle content.

## 2. Materials and Methods

### 2.1. Matrix

**Figure 1:**
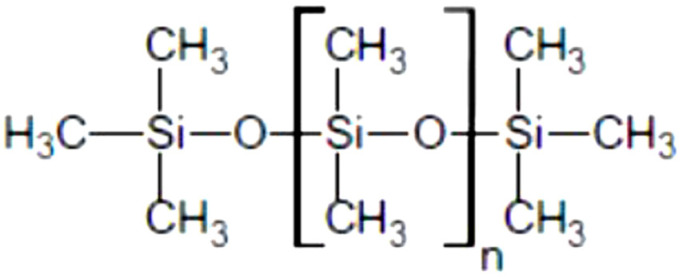
The structure of silicon rubber.

Vulcanized silicone rubber was used as a flexible matrix material. The most common form of silicone is the polydimethylsiloxane polymer, which is liquid in origin. This polymer is a rigid structure of elastomers transformed by catalyzed cross-linking reactions [22]. To obtain catalyzed cross-linking reactions, a stiffener with 4% (by weight) must be added to the silicon rubber. The specific gravity of SR was 1.12 g/mL and the elongation was 350%. The main elements of SR are hydrogen, carbon, oxygen and silicon, as shown in Figure 1.

### 2.2. Fillers

**Figure 2:**
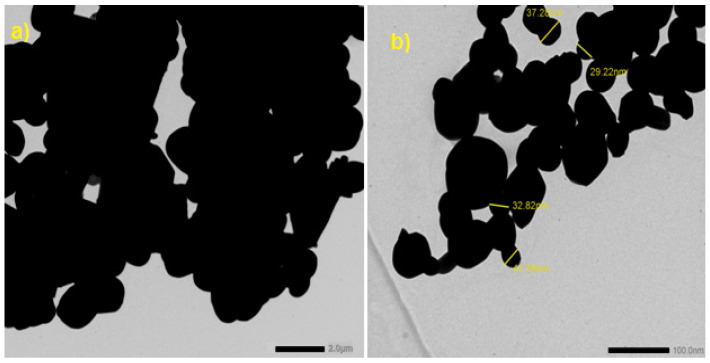
TEM images for (**a**) Bi_2_O_3_ microparticles and (**b**) Bi_2_O_3_ nanoparticles.

Bismuth oxide (Bi_2_O_3_) of micro and nano sizes was used as a filler in the composite. Before adding it to the solution, a transmission electron microscope (TEM) is used to image the powder to ensure the size of the particles, as shown in Figure 2. The average size of the microparticles was 15 ± 5 µm, while the average size of the nanoparticles was 30 ± 5 nm.

### 2.3. Composites

Seven different SR samples were prepared. Codes, compositions and densities of the prepared samples are tabulated in Table 1. The homogenous mixtures (liquid SR + micro- or nanoparticles of Bi_2_O_3_ + stiffener) were poured into cylinder molds, which had a 3 cm diameter and different thicknesses (0.3, 0.66, 0.93 and 1.3 cm). The prepared samples waited for 24 h to become elastic-solid materials. The density of the SR sample is measured via the mass/volume, the volume of the sample is calculated by ($\pi r^{2}\cdot x$), where r is the radius and x is the thickness of the measured sample.

### 2.4. Morphological Images

A scanning electron microscope (SEM) of JSM-5300, JEOL model, Tokyo, Japan was used for scanning the images of the prepared SR samples. The samples were coated using an ion sputtering coating device (JEOL-JFC-1100E, Tokyo, Japan), and then the samples were placed inside the electron microscope with an operating voltage of 20 keV [23].

### 2.5. Mechanical Properties

The tensile strength, Young’s modulus and elongation at break were determined for the present SR samples using an electronic tensile testing machine (model 1425, Germany), according to standard methods with ASTM D412. The Shore hardness was measured according to ASTM D2240.

### 2.6. Shielding Properties

**Figure 3:**
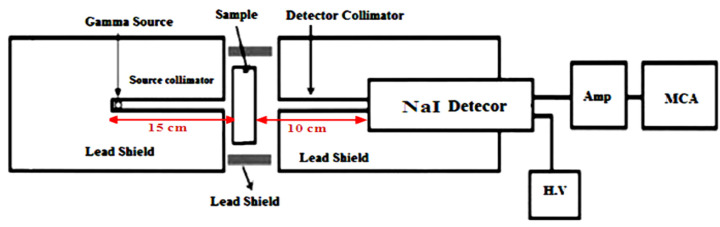
The experimental setup used to measure the attenuation coefficient.

The linear attenuation coefficient (*LAC*) was measured for all discussed SR samples using the narrow beam technique of gamma ray spectroscopy in a radiation physics laboratory (Faculty of Science, Alexandria University, Alexandria, Egypt). The devices used in this method were the detector, collimator and radioactive point sources. An NaI (Tl) cylindrical scintillation detector with a (3″ × 3″) dimension, a relative efficiency of 15% and an energy value of Cs-137 (0.662 MeV) was used. The inner diameter of the lead collimator was 8mm and the outer diameter was 100 mm. The point radioactive sources were chosen to cover a wide range of energy, where four radioactive sources were used as follows: Am-241 (0.06 MeV), Ba-133 (0.081, 0.356 MeV), Cs-137 (0.662 MeV) and Co-60 (1.173, 1.333 MeV) [24,25,26,27,28,29]. The illustration of the experimental setup is shown in Figure 3.

The intensity (I_0_), count rate ($N_{0}$) or area under the peak ($A_{0}$) were measured for all energies in a case without the SR sample, and then the sample was placed between the source and detector and the count rate (N) or area under the peak (A) was measured at the same time. The *LAC* was measured experimentally using the following equation [30,31]:

(1) $$LAC=\frac{1}{x}\;ln\frac{N_{0}}{N}=\frac{1}{x}\;ln\frac{A_{0}}{A}$$

The experimental values of the LAC for SR and the micro filler were compared to the results obtained from the XCOM software [32,33]. The relative deviation between the two results is calculated by the following:

(2) $$Dev\left(\%\right)=\frac{LAC_{xcom}-LAC_{exp}}{\;LAC_{exp}}\times 100$$

While the relative increase between the results of the *LAC* of the micro and nano fillers are evaluated via the following:

(3) $$R\cdot I\left(\%\right)=\frac{LAC_{nano}-LAC_{micro}}{\;LAC_{micro}}\times 100$$

The other radiation attenuation parameters are based on the *LAC*, such as the Half Value Layer (*HVL*), which represents the thickness needed to reduce the initial intensity to its half value; the Mean Free Path (*MFP*), which represents the path of the photon inside the sample without any interactions; and the Tenth Value Layer (*TVL*), which represents the thickness needed to reduce the initial intensity to its tenth value and can be estimated from the following equation [34,35,36]:

(4) $$HVL=\frac{\ln2}{LAC},\;\;\;\;MFP=\frac{1}{LAC},\;\;\;TVL=\frac{\ln10}{LAC}$$

The efficiency of shielding materials is estimated by an important parameter called the Radiation Protection Efficiency (*RPE*) and calculated using the following equation [37,38,39]:

(5) $$RPE\left(\%\right)=\left[1-\frac{N}{N_{0}}\right]\times 100$$

## 3. Results and Discussion

### 3.1. SEM Results

**Figure 4:**
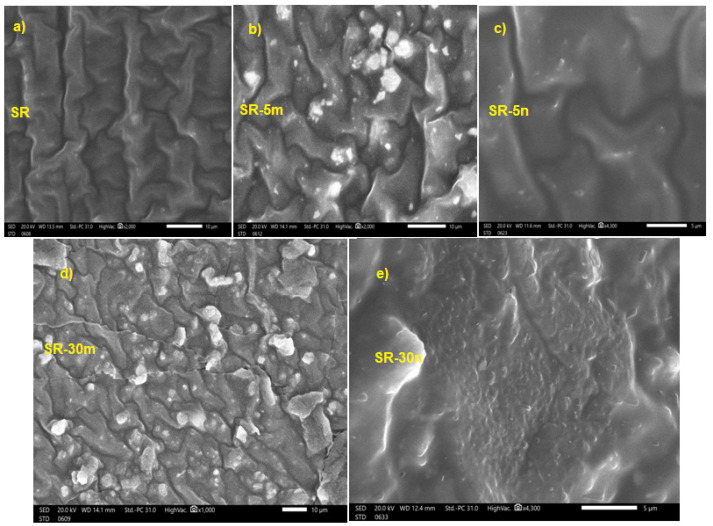
SEM images of the prepared samples of (**a**) SR, (**b**) SR-5m, (**c**) SR-5n, (**d**) SR-30m and (**e**) SR-30n.

SEM images of the prepared samples were scanned and showed, in general, a good distribution of Bi_2_O_3_ with the SR composite. On the other hand, the distribution of nanoparticles was better than the microparticles, as shown in Figure 4, which means that the SR containing nanoparticles of Bi_2_O_3_ has a higher surface area and lower porosity, compared to the same contents of SR containing microparticles of Bi_2_O_3_. as Additionally, the porosity of SR containing nanoparticles of Bi_2_O_3_ is low, which led to an increase in the mechanical and shielding properties.

### 3.2. Mechanical Results

**Figure 5:**
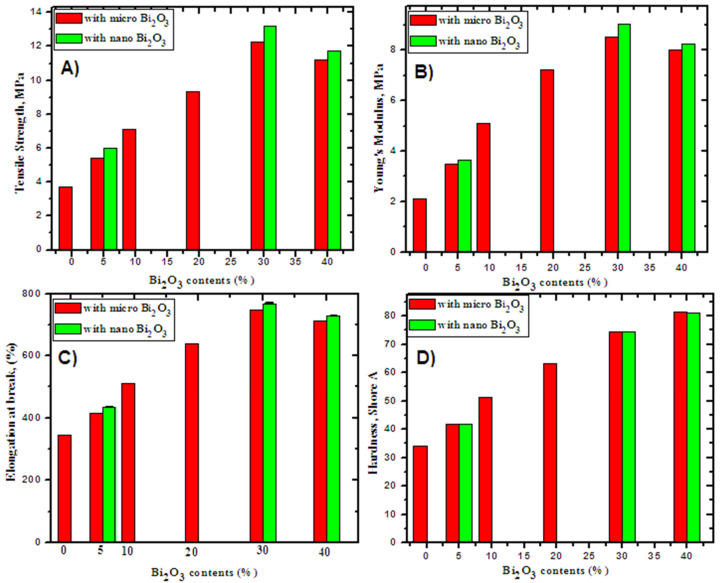
The mechanical properties of SR with different contents of micro- and nano-Bi_2_O_3_. (**A**) Tensile Strength, MPa, (**B**) Young modulus, MPa, (**C**) Elongations (%), (**D**) Hardness, Shore A.

In the case of free SR, the mechanical properties (MPs) of SR composites are relatively poor. The MPs of the SR/micro- and nano Bi_2_O_3_ composites are plotted in Figure 5A–D. These figures show the variability of tensile strength, Young’s modulus and elongation at break with different concentrations of micro- and nano-Bi_2_O_3_ as fillers. The results show that increasing the filler load leads to a significant increase in the tensile strength, Young’s modulus and elongation at break of up to 30 wt%.

The results also show that the addition of nano-Bi_2_O_3_ produces a greater an increase in tensile strength, Young’s modulus and elongation at break than micro-Bi_2_O_3_ with the same percentage. The concentration of the filler was increased to 40%, and the same mechanical properties were studied as before, and it was found that it was less than 30%, and this is what made us conduct a comprehensive study with a maximum of 30% for micro- and nano-Bi_2_O_3_ as a filler in the SR. Low mechanical properties at 40 wt% of the filler content is likely due to the accumulation of filler material in different rubber layers. The hardness was increased with the increase in the filler contents, and this was normal because the addition of filler in the SR leads to an increase in material hardness.

### 3.3. Shielding Results

**Figure 6:**
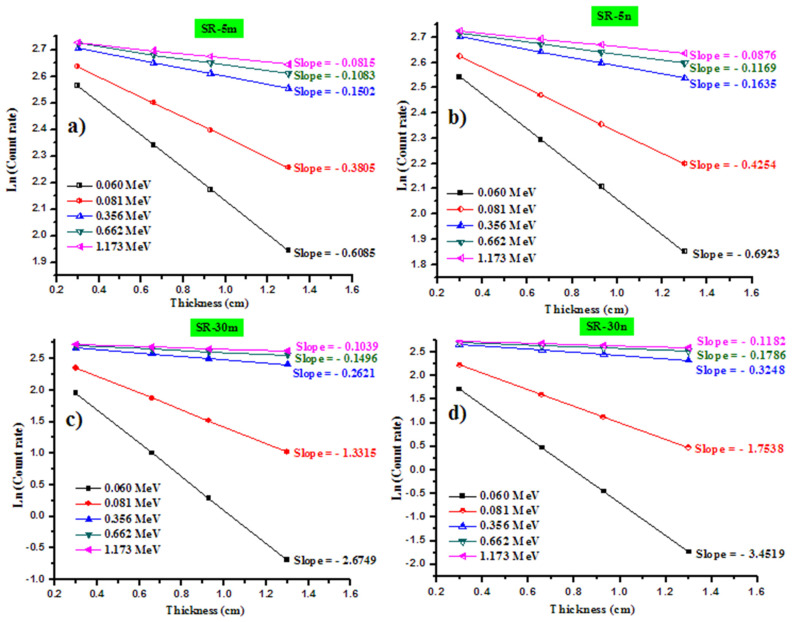
Graphical representation of the reduction in the intensity of the photons as a function of the thickness for (**a**) SR-5m, (**b**) SR-5n, (**c**) SR-30m and (**d**) SR-30n.

In order to obtain the linear attenuation coefficient experimentally, we represented the relation between Ln (I/I_0_) and the thickness of the samples, according to the Lambert–Beer law. The slope of the straight line is the absolute value of the *LAC*. We represent the reduction in the intensity of the photons as a function of the thickness for four samples in Figure 6a–d. In this figure, we show the results for the following samples: SR-5m, SR-5n, SR-30m and SR-30n. The other samples have the same trend shown in this figure, so we did not present the data for the remaining samples in Figure 6a–d.

As one can see in this figure, the slope is negative, which implies that the transmission of the photons through the prepared silicone rubber samples decrease with increasing the thickness from 3 to 13 mm. The slope of the rubber silicon sample, SR-5m, is −0.1502 at 0.356 MeV, and, as can be seen from Table 2, the *LAC* for this sample at 0.356 MeV is 0.1502 cm^−1^. The *LAC* values for all the prepared rubber silicon with different amounts of micro- and nano-Bi_2_O_3_ is summarized in Table 3.

In this study, the linear attenuation coefficient (*LAC*) values of the SR without Bi_2_O_3_ and with 5, 10, 20 and 30% Bi_2_O_3_ (in micro and nano sizes) were experimentally measured using different radioactive point sources in the energy range varying from 0.06 to 1.333 MeV. Additionally, we theoretically calculated the *LAC* for the SR with micro-Bi_2_O_3_ using XCOM software. The other parameters based on *LAC* were calculated, such as HVL, MFP and TVL, and tabulated in Table 4.

**Figure 7:**
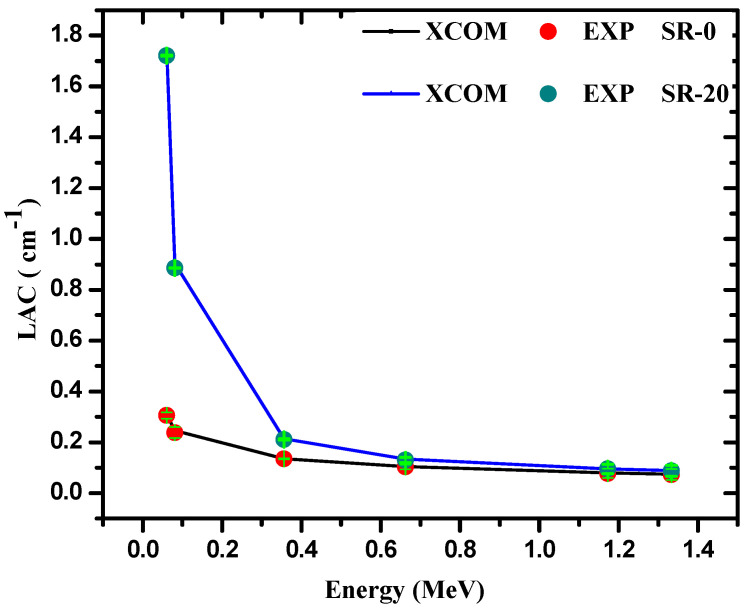
Comparison between the experimental and XCOM *LAC* for the SR 0 and 20% of micro-PnO.

The comparison between the experimental and theoretical *LAC* for the SR (free Bi_2_O_3_) and SR with 20% micro-Bi_2_O_3_ is plotted in Figure 7. We notice good comparability between both *LAC* values measured in the lab and those calculated by XCOM. This is true for most tested energies, however, we found some minor differences between both approaches, and this is acceptable since usually anyone can find some small errors in the experimental part, but generally the experimental results match the XCOM results in an acceptable manner. This is an essential and important step since it provides confidence in the accuracy of the geometry utilized in the lab for the determination of *LAC* for the SR and SR/micro-Bi_2_O_3_ samples. We also calculated the error (Dev.%) between the experimental and XCOM data.

We found that the Dev.% for SR (free Bi_2_O_3_) is confined between 0.25 and 2.55%, while the Dev.% for the SR with 10 micro-Bi_2_O_3_ is limited between 0.62 and 3.25%. The Dev.% also ranges between 0.28 and 2.14% for the SR with 20 micro-Bi_2_O_3_, while the Dev.% for the SR with 30 micro-Bi_2_O_3_ is limited between 1.22% and 2.54%. These results confirm that the Dev.% is small (less than 3%), which reaffirms the compatibility of the practical and theoretical results.

**Figure 8:**
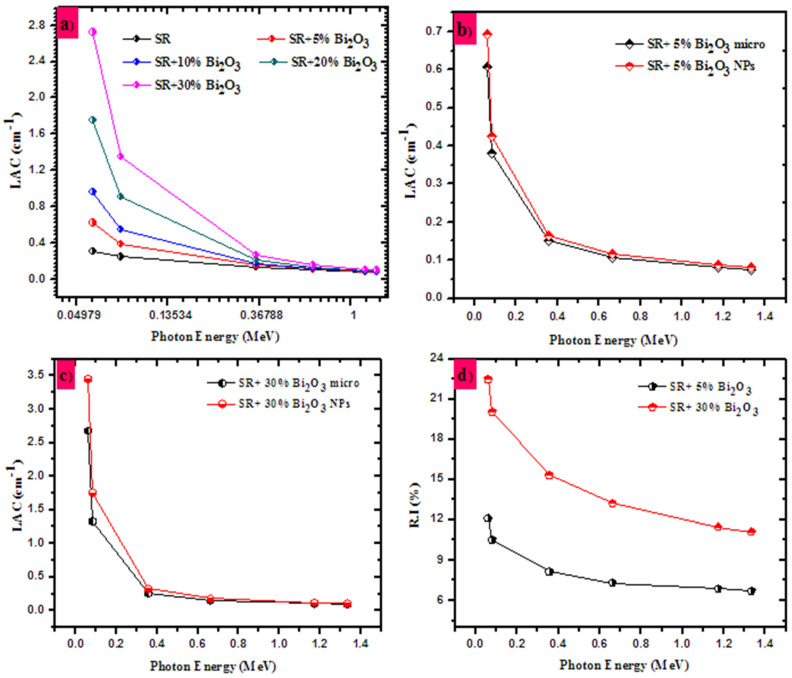
The linear attenuation coefficient for the SR (**a**) with 0, 5, 10, 20 and 30 micro-Bi_2_O_3_, (**b**) with 5% of micro- and nano-Bi_2_O_3_, (**c**) with 30% of micro- and nano-Bi_2_O_3_, and (**d**) the relative increase in the *LAC*.

In Figure 8a, we plot the *LAC* for the SR and the RS with different concentrations of micro-Bi_2_O_3_ (5, 10, 20 and 30%). Using this figure, we aim to understand the influence of adding some fractions of Bi_2_O_3_ into the SR on the attenuation performance of the prepared samples. Evidently, the lowest *LAC* is found in the SR and the *LAC* progressively increases as the amount of Bi_2_O_3_ increases from 5 to 30%, where the maximum *LAC* is reported for the SR + 30% Bi_2_O_3_ sample. The reason for this enhancement in *LAC* is due to the high density and atomic number of bismuth, and it is known that adding high atomic number elements to the materials increases the probability of the interaction between the photons and the electrons in the materials. Consequently, incorporating Bi_2_O_3_ into the SR sample leads to the enhancement of the radiation protection performance.

In order to compare the effect of Bi_2_O_3_ size on the attenuation performance of the SR, we plotted the *LAC* for the SR with 5% micro- and nano-Bi_2_O_3_ in Figure 8b, and SR with 30% micro- and nano-Bi_2_O_3_ in Figure 8c. The *LAC* values for the SR-5n is higher than the *LAC* of SR-5m, and the same for 30% (i.e., the *LAC* for the SR with nanoparticles is higher than micro-Bi_2_O_3_). These results imply that the radiation interaction probability increases when the micro-Bi_2_O_3_ is replaced with nano-Bi_2_O_3_ in the SR. From Equation (3), we define a parameter called relative increase (R.I), which shows the enhancement in the *LAC* due to the replacement of micro-Bi_2_O_3_ by nano-Bi_2_O_3_. The R.I is higher than 1, which reaffirms the importance of using nanosized Bi_2_O_3_ to develop an effective attenuation barrier (see Figure 8d). Additionally, the RI for 30% of Bi_2_O_3_ is higher than that with 5% of Bi_2_O_3_. Accordingly, SR with 30% of Bi_2_O_3_ has interesting radiation shielding features, compared to the SR with micro-Bi_2_O_3_.

**Figure 9:**
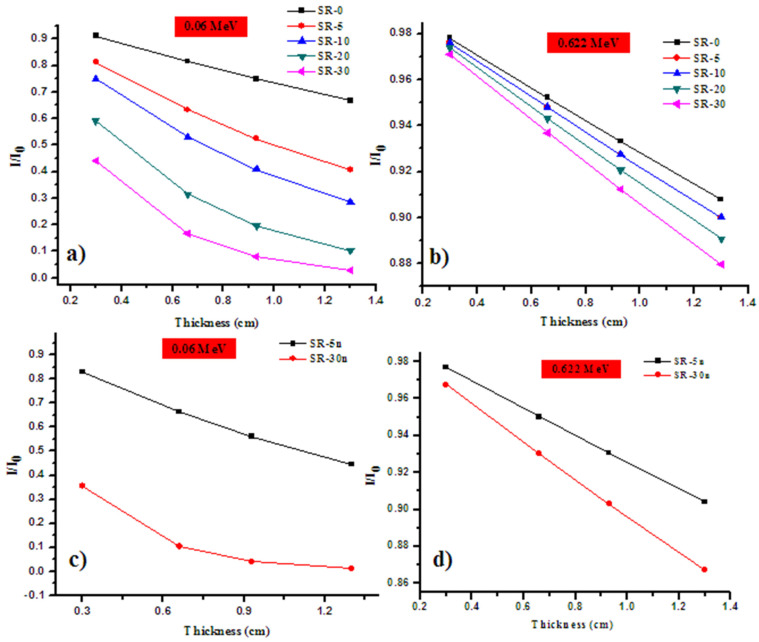
The measured gamma photon transmission through the RS with micro-Bi_2_O_3_ and nano-Bi_2_O_3_, respectively, (**a**) RS with micro-Bi_2_O_3_ at 0.06 MeV, (**b**) RS with micro-Bi_2_O_3_ at 0.662 MeV, (**c**) RS with nano-Bi_2_O_3_ at 0.06 MeV and (**d**) RS with nano-Bi_2_O_3_ at 0.662 MeV.

Figure 9a,b shows the measured gamma photon transmission through the RS with micro-Bi_2_O_3_ and nano-Bi_2_O_3_, respectively. We call this T micro and T nano. It can be seen that both T micro and T nano exponentially decrease with increasing the thickness from 3 to 13 mm. In Figure 9a, the T micro is lower than that of SR (free Bi_2_O_3_), which means that the transmission of the photons through the sample with Bi_2_O_3_ is lower than the transmission of photons through the free Bi_2_O_3_ SR sample. This means that the addition of Bi_2_O_3_ reduces the transmission of the photons through the prepared silicon rubber. Additionally, we can see that the T micro depends on the amount of Bi_2_O_3_ incorporated into the SR. The more Bi_2_O_3_ in the SR, the lower the T micro. Hence, the incorporation of Bi_2_O_3_ has a positive influence on the attenuation performance of the SR. If we observe Figure 9b, we can conclude the same results obtained in Figure 9a. In the other words, the photon’s transmission through free Bi_2_O_3_-SR is higher than the SR with nano-Bi_2_O_3_. This result reaffirms that the increase in the weight fraction of Bi_2_O_3_ in the SR can efficaciously diminish the photon’s transmittance. Therefore, a high amount of Bi_2_O_3_ (micro or nano sized) in the SR is a good choice to improve the gamma ray shielding performance for the prepared SR.

**Figure 10:**
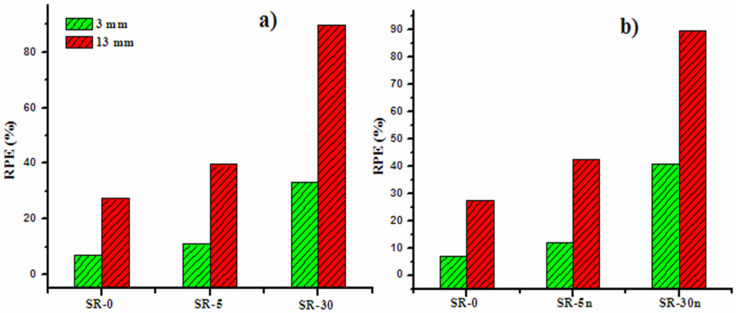
The measured radiation protection efficiency at 3 and 13 mm through (**a**) the RS with micro-Bi_2_O_3_ and (**b**) nano-Bi_2_O_3_ at 0.081 MeV.

In order to understand the influence of the thickness of the prepared SR on the attenuation performance, we plotted the radiation shielding efficiency (RPE) for the SR, SR-5m and SR-30m, in Figure 10a, and SR, SR-5n and SR-30n, in Figure 10b, at the same energy value of 0.081 MeV. In both subfigures, it is evident that the RPE for the SR is less than the RPE for the SR with Bi_2_O_3_ (micro or nano sized), which reaffirms that adding Bi_2_O_3_ to the SR causes an improvement in the attenuation performance. Most importantly, we can see that the RPE for the SR with a thickness of 13 mm is higher than that with a thickness of 3 mm. This is correct for the SR incorporating micro- or nano-Bi_2_O_3_. For instance, from Figure 10a, for the SR-5m, the RPE is 10% and this is increased to 40% for the same sample with a thickness of 13 mm. Therefore, we can conclude that the thickness is an important parameter that affects the attenuation competence of the prepared SR. The high thickness SR is recommended as a good attenuator barrier. Moreover, we found that the RPE for the SR with nano-Bi_2_O_3_ is higher than that of the corresponding micro-Bi_2_O_3_.

**Figure 11:**
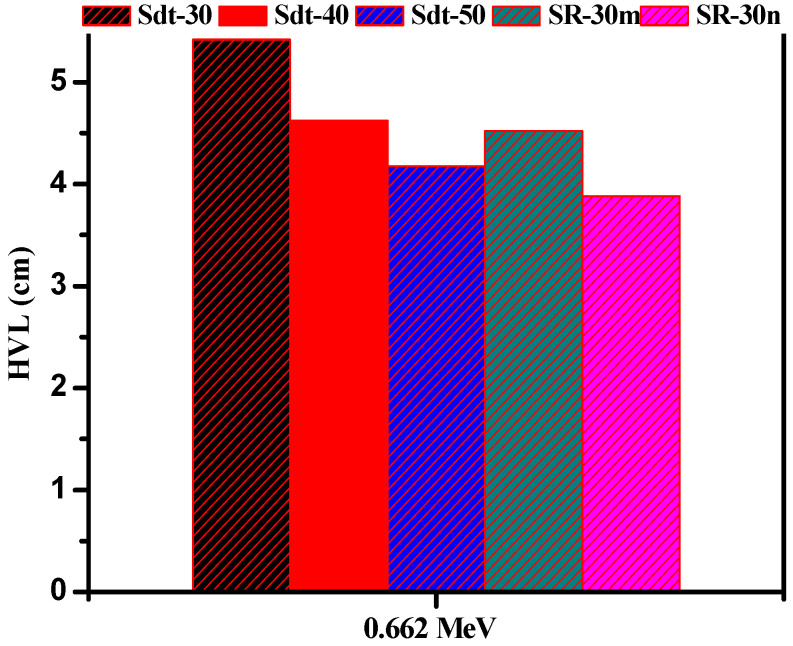
The HVL of 30% micro and nano prepared samples compared with SR filled with magnetite iron.

It is important to compare the radiation attenuation performance for the prepared SR doped with Bi_2_O_3_ with a similar material, in order to check the possibility of using these samples in real applications. For this purpose, we compared the half value layer of the SR with 30% of micro- and nano-Bi_2_O_3_ with 3 samples: SR 30, 40 and 50% of magnetite iron [40], as shown in Figure 11. We selected one energy value in the comparison, i.e., 0.662 MeV. Evidently, the SR with 30% of Bi_2_O_3_ (micro and nano sized) have a lower HVL and thus better attenuation competence than the SR with 30% of magnetite iron. The SR with 30% of micro-Bi_2_O_3_ has an HVL of 4.52 cm and this is close to 4.62 cm, which was reported for the SR with 40% of iron. The SR with 30% nano-Bi_2_O_3_ is lower than that of the SR with 30 and 40% of iron, but has an almost similar HVL, with the SR being in contact with 50% of the magnetite iron.

## 4. Conclusions

Flexible materials were prepared from SR and different sizes of Bi_2_O_3_. The morphological, mechanical and shielding properties were determined. The SEM results indicate that the nano filler is significantly better than micro filler. The mechanical results conclude that the flexibility of the materials decreases as we increase the Bi_2_O_3_ filler with 30 wt%. Therefore, the attenuation study was controlled by the flexibility results. The *LAC* was determined experimentally and the results show good agreement with the theoretical results. The attenuation coefficients of the prepared SR samples showed a clear superiority in lower energy levels over other energies, and the SR’s nano-Bi_2_O_3_ was better than the corresponding SR’s micro-Bi_2_O_3_ at all discussed energies for the shielding materials.

## Figures and Tables

**Table 1:** Codes, compositions and densities of the prepared SR samples.

Code	Compositions (wt%)				Density (g/cm^3^)
	SR	Micro-Bi_2_O_3_	Nano-Bi_2_O_3_	Stiffener	
SR-0	100	-	-	4	1.191
SR-5m	95	5	-		1.301
SR-5n	95	-	5		1.351
SR-10m	90	10	-		1.368
SR-20m	80	20	-		1.509
SR-30m	70	30	-		1.684
SR-30n	70	-	30		1.713

**Table 2:** Linear attenuation coefficient of silicon rubber with different additives (fraction by weight).

Energy (MeV)	SR-0			SR-10m			SR-20m		
	XCOM	EXP	Dev (%)	XCOM	EXP	Dev (%)	XCOM	EXP	Dev (%)
0.060	0.3097	0.3059	1.25	0.9620	0.9464	1.65	1.7499	1.7215	1.65
0.081	0.2456	0.2384	3.01	0.544	0.5305	2.52	0.9042	0.8860	2.05
0.356	0.1354	0.1351	0.25	0.171	0.1657	3.25	0.2141	0.2113	1.35
0.662	0.1043	0.1033	0.98	0.118	0.1154	1.85	0.1336	0.1308	2.14
1.173	0.0794	0.0779	1.89	0.087	0.0861	0.62	0.0954	0.0952	0.28
1.333	0.0744	0.0728	2.11	0.081	0.0796	1.63	0.0889	0.0876	1.48

**Table 3:** Linear attenuation coefficient of bulk and nano samples.

Energy (MeV)	SR-5					SR-30				
	XCOM	SR-5m	Dev (%)	SR-5n	R.I (%)	XCOM	SR-30m	Dev (%)	SR-30m	R.I (%)
0.060	0.6213	0.6085	2.11	0.6923	12.11	2.7206	2.6749	1.71	3.4519	22.51
0.081	0.3881	0.3805	1.99	0.4254	10.55	1.3481	1.3315	1.25	1.7538	20.08
0.356	0.1525	0.1502	1.52	0.1635	8.14	0.2672	0.2621	1.95	0.3248	15.32
0.662	0.1106	0.1083	2.15	0.1169	7.31	0.1534	0.1496	2.54	0.1786	13.22
1.173	0.0829	0.0815	1.62	0.0876	6.88	0.1063	0.1039	2.31	0.1182	11.46
1.333	0.0775	0.0756	2.55	0.0810	6.67	0.0987	0.0976	1.22	0.1098	11.11

**Table 4:** The HVL, MFP and TVL for all the micro and nano prepared samples.

Shielding Parameters	Energy (MeV)	SR-0	SR-5m	SR-5n	SR-10m	SR-20m	SR-30m	SR-30n
HVL (cm)	0.060	2.2380	1.1156	1.0012	0.7206	0.3961	0.2548	0.2008
	0.081	2.8221	1.7860	1.6294	1.2745	0.7666	0.5142	0.3952
	0.356	5.1177	4.5465	4.2399	4.0525	3.2373	2.5943	2.1339
	0.662	6.6456	6.2649	5.9318	5.8966	5.1878	4.5185	3.8818
	1.173	8.7318	8.3659	7.9165	8.0008	7.2628	6.5214	5.8647
	1.333	9.3223	8.9437	8.5600	8.5649	7.7959	7.0193	6.3156
MFP (cm)	0.060	3.2287	1.6095	1.4444	1.0395	0.5715	0.3676	0.2897
	0.081	4.0714	2.5767	2.3507	1.8387	1.1060	0.7418	0.5702
	0.356	7.3833	6.5592	6.1169	5.8466	4.6705	3.7428	3.0786
	0.662	9.5876	9.0383	8.5577	8.5069	7.4844	6.5188	5.6002
	1.173	12.5973	12.0695	11.4212	11.5427	10.4779	9.4084	8.4610
	1.333	13.4492	12.9030	12.3495	12.3565	11.2472	10.1267	9.1114
TVL (cm)	0.060	7.4345	3.7060	3.3259	2.3936	1.3158	0.8463	0.6670
	0.081	9.3748	5.9330	5.4126	4.2338	2.5466	1.7080	1.3129
	0.356	17.0007	15.1031	14.0846	13.4623	10.7542	8.6181	7.0887
	0.662	22.0762	20.8115	19.7049	19.5879	17.2335	15.0102	12.8949
	1.173	29.0063	27.7910	26.2982	26.5780	24.1264	21.6636	19.4822
	1.333	30.9680	29.7103	28.4357	28.4519	25.8976	23.3176	20.9799

## Data Availability

The data presented in this study are available on request from the corresponding author.
